# K63 linked ubiquitin chain formation is a signal for HIF1A degradation by Chaperone-Mediated Autophagy

**DOI:** 10.1038/srep10210

**Published:** 2015-05-11

**Authors:** Joao Vasco Ferreira, Ana Rosa Soares, Jose Silva Ramalho, Paulo Pereira, Henrique Girao

**Affiliations:** 1 Center of Ophthalmology and Vision Sciences; Institute for Biomedical Imaging and Life Science (IBILI); Faculty of Medicine; University of Coimbra; Coimbra, Portugal; 2 CEDOC; Faculty of Medicine, New University of Lisbon; Lisbon; Portugal

## Abstract

Chaperone-Mediated Autophagy is a selective form of autophagy. Recently, the degradation of a newly identified CMA substrate, the HIF1A transcription factor, was found to be regulated by the ubiquitin ligase STUB1. In this study we show, for the first time, that K63 ubiquitination is necessary for CMA degradation of HIF1A *in vitro* and *in vivo*. Additionally, STUB1 mediates K63 linked ubiquitination of HIF1A. Our findings add a new regulatory step and increase the specificity of the molecular mechanism involved in CMA degradation of HIF1A, expanding the role of ubiquitination to yet another biological process, since the same mechanism might be applicable to other CMA substrates.

The Hypoxia-Inducible Factor 1 (HIF1) transcription factor is a heterodimer composed of two subunits: hypoxia inducible factor 1, α subunit (basic helix-loop-helix transcription factor) (HIF1A/HIF-1α) and aryl hydrocarbon receptor nuclear translocator (ARNT/HIF-1β)^1^,^2^. When oxygen is available, HIF1A is hydroxylated by specific prolyl hydroxylases^3^,^4^ and recognized by the von Hippel-Lindau tumor suppressor (VHL) protein that targets HIF1A for polyubiquitination and subsequent degradation by the proteasome^5^. When oxygen becomes limiting the protein escapes degradation, HIF1A dimerizes with ARNT and promotes the expression of numerous hypoxia-responsive genes that are critical in ensuring cell survival under low oxygen^6^. However, recent evidences indicate that the degradation of HIF1A may also occur through the lysosome, in an O_2_-independent manner, by Chaperone-Mediated Autophagy (CMA), a selective type of autophagy^7^,^8^. However, the molecular mechanisms involved in CMA-mediated degradation of the transcription factor are yet to be fully elucidated.

In the Ubiquitin-Proteasome System (UPS), recognition of substrates involves their tagging by ubiquitin, through a process mediated by the ubiquitin conjugation machinery^9^. This process can be repeated several times, by the attachment of additional ubiquitin moieties to one of the 7 Lysine (K) residues present in ubiquitin, resulting in the formation of different types of linear or branched polyubiquitin chains. The topology of ubiquitin chains is often associated with different biological roles. For example, polyubiquitin chains formed through K48 are usually associated with the targeting of proteins to the proteasome^10^. On the other hand, K63 linked chains have been involved in DNA repair^11^, internalization of plasma membrane proteins^12^,^13^,^14^, protein sorting to multivesicular bodies^15^,^16^,^17^ and protein and/or subcellular organelles degradation in the lysosome by macroautophagy^18^,^19^,^20^.

Although the selectivity of proteasomal degradation is conferred by ubiquitination, in the case of the lysosome, the selectivity can reside on ubiquitination and/or on motifs present in substrates that target them for degradation. For example, in the case of CMA, substrate selectivity is conferred by a targeting motif biochemically related to the pentapeptide KFERQ, which is recognized by HSC70 (heat shock 70 kDa protein 8), that subsequently directs the substrate to the lysosome^21^. At the lysosomal membrane, substrates interact with lysosomal-associated membrane protein 2A (LAMP2A)^21^ which acts as the CMA receptor, mediating the translocation of substrates to the lumen of the lysosome. Previous studies from our lab demonstrated that the ubiquitin ligase STUB1 is required for degradation of HIF1A by CMA^7^,^22^, which constitutes the first evidence that ubiquitination can target substrates to CMA. However, the molecular mechanisms whereby ubiquitin signals a substrate to CMA are yet to be fully elucidated. STUB1 is able to interact with major cytoplasmic chaperones HSC70, HSP70, and HSP90 and ubiquitinate substrates that are bound to those chaperones. It has been established that ubiquitination mediated by STUB1 can target substrates to the proteasome^23^,^24^,^25^ or the lysosome^7^,^26^,^27^, suggesting a pivotal role for this E3 ligase in mediating triage decisions, regarding substrate degradation, between the proteasomal or the lysosomal systems. Nonetheless, thus far, a putative role for ubiquitin modification of substrates in CMA remained largely unexplored.

In this study we establish, for the first time, that K63 polyubiquitin chains are required for degradation of HIF1A by CMA. Moreover, we demonstrate that K63 ubiquitination of HIF1A is mediated by the ubiquitin ligase STUB1 and is dependent on the KFERQ–like consensus sequence present in the protein. We further show that starvation induces K63 ubiquitin conjugation to HIF1A *in vivo*.

## Results

### Inhibition of K63 ubiquitination blocks HIF1A degradation by CMA

Although we previously showed that degradation of HIF1A by CMA is dependent on the ubiquitin ligase STUB1^7^, the ubiquitination of HIF1A as a signal for degradation by CMA remains to be elucidated. To address this issue we used two different cell lines, the human cervical cancer HeLa cells and the mouse embryonic fibroblasts NIH-3T3 cells. First we evaluated whether, in our experimental conditions, HIF1A was a substrate for CMA (S.1A, B, C, D and E). Having established that both HeLa and NIH-3T3 cells can degrade HIF1A by CMA, we investigated the role of ubiquitin signaling in this process. For this purpose, cells were transfected with plasmids that encode ubiquitin mutants in which either lysine 48 or 63 are substituted by an arginine, to specifically inhibit the formation of either lysine 48 chains (K48R) or lysine 63 chains (K63R). Furthermore, we also used mutants that restrict ubiquitination to only one kind of ubiquitin chain. In this case, all the lysines of ubiquitin are substituted by arginine but lysine 48 (K48) or 63 (K63). Since under normoxic conditions HIF1A is a very labile protein, virtually undetectable at normal oxygen concentrations^28^, all the immunoprecipitation experiments were performed in cells incubated with CoCl_2_, shown to inhibit HIF1A hydroxylation and subsequent VHL-dependent K48 ubiquitination of HIF1A. This allows the stabilization of the protein while maintaining ubiquitin conjugation mediated by other E3s.

**Figure 1:**
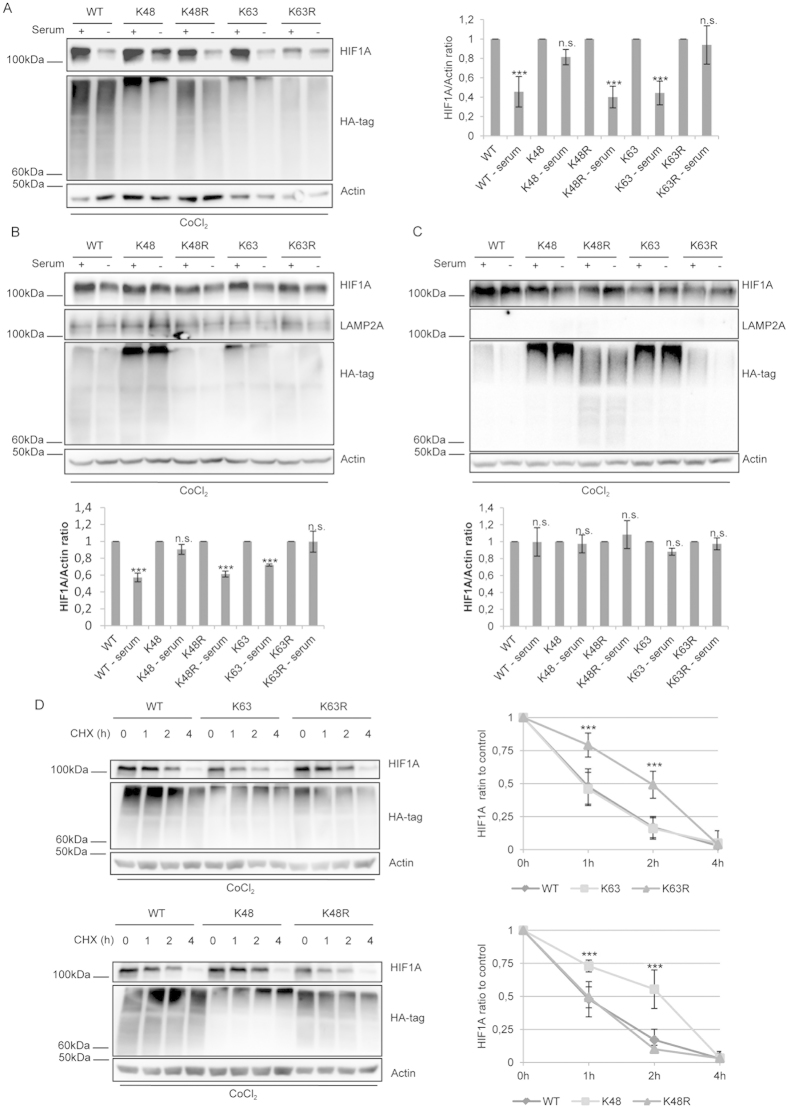
K63 ubiquitin linked chains are necessary for CMA-mediated degradation of HIF1A. Cells were transfected with plasmids for either wild-type or mutant ubiquitins tagged with hemagglutinin (HA) and left overexpressing for 48 h. NIH-3T3 cells were transduced with empty or shRNA containing lentiviral vectors against LAMP2A. After, cells were incubated in the presence or absence of serum, 300 uM of CoCl_2_, 2% O_2_ and 50 ug/ml of CHX (**A, B and C**). In HeLa and NIH-3T3 cells incubated with CoCl_2_ (**A,B**) overexpression of the K48 and K63R mutants, but not of the K48R and K63 mutants, inhibits the degradation of HIF1A after 6 h of serum removal. (**C**) In LAMP2A depleted cells, HIF1A protein levels are unchanged after serum removal both in wild-type and mutant ubiquitin overexpressing cells. (**D**) HeLa cells were pre-incubated with CoCl_2_ for two hours to stabilize HIF1A. After, cells were incubated with CHX for the indicated times while CoCl_2_ was maintained for the remaining of the experiment. The overexpression of the K63R and K48 mutants, but not of the K63 and K48R mutants, increases the half-life of HIF1A from 1 to 2 h. The blots used in the figure are cropped. All the gels have been run under the same experimental conditions. The results represent the mean ±SD of at least three independent experiments (n.s. nonsignificant; *p < 0.05; **p < 0.01; ***p < 0.001).

Both in Hela and NIH-3T3 cells incubated with CoCl_2_, overexpression of the K48 mutant, but not of the K48R mutant, prevented HIF1A degradation induced by serum removal (Fig. 1A,B), suggesting that K48 linked ubiquitin chains are not required for HIF1A degradation by CMA. On the other hand, the K63R, but not the K63, mutant overexpression hindered the decrease of HIF1A protein levels after serum removal (Fig. 1A,B). In Hela cells, after serum removal at 2% O_2_, inhibition of K63 linked ubiquitin conjugation results in the stabilization of HIF1A, when compared to the wild-type (S1.F).

To specifically inhibit CMA we depleted the lysosomal protein LAMP2A in NIH-3T3 cells by using lentiviral vectors encoding a shRNA^7^. The results presented in Fig. 1B show that both wild-type or mutant ubiquitin overxpression failed to decrease HIF1A protein levels after serum removal.

To further confirm the specificity of K63 ubiquitin chain conjugation in HIF1A degradation by CMA, we overexpressed lysine 29 based mutants as a control. Data was similar to that of the lysine 48 based mutants, where overexpression of the K29 mutant, but not of the K29R mutant, inhibited the decrease of HIF1A protein levels after serum removal (S2.A). Next, we evaluated the involvement of K63 linked ubiquitin chains in HIF1A turnover, under basal conditions, when CMA is not stimulated. For this purpose we inhibited protein synthesis using cycloheximide (CHX) for different periods of time, in the presence of CoCl_2_. In accordance, overexpression of the K63R or K48 mutants increased HIF1A half-life from 1 h to 2h approximately (Fig. 1E), while the other mutants failed to do so. Also, in cells incubated at 2% O_2_ and 2h of CHX, inhibition of K63 linked ubiquitin conjugation results in the stabilization of HIF1A, when compared to the wild-type (S.2B). Altogether, these results indicate that K63 linked ubiquitin chains are needed for HIF1A degradation by CMA.

### HIF1A is conjugated with K63 linked ubiquitin chains after CMA activation

**Figure 2:**
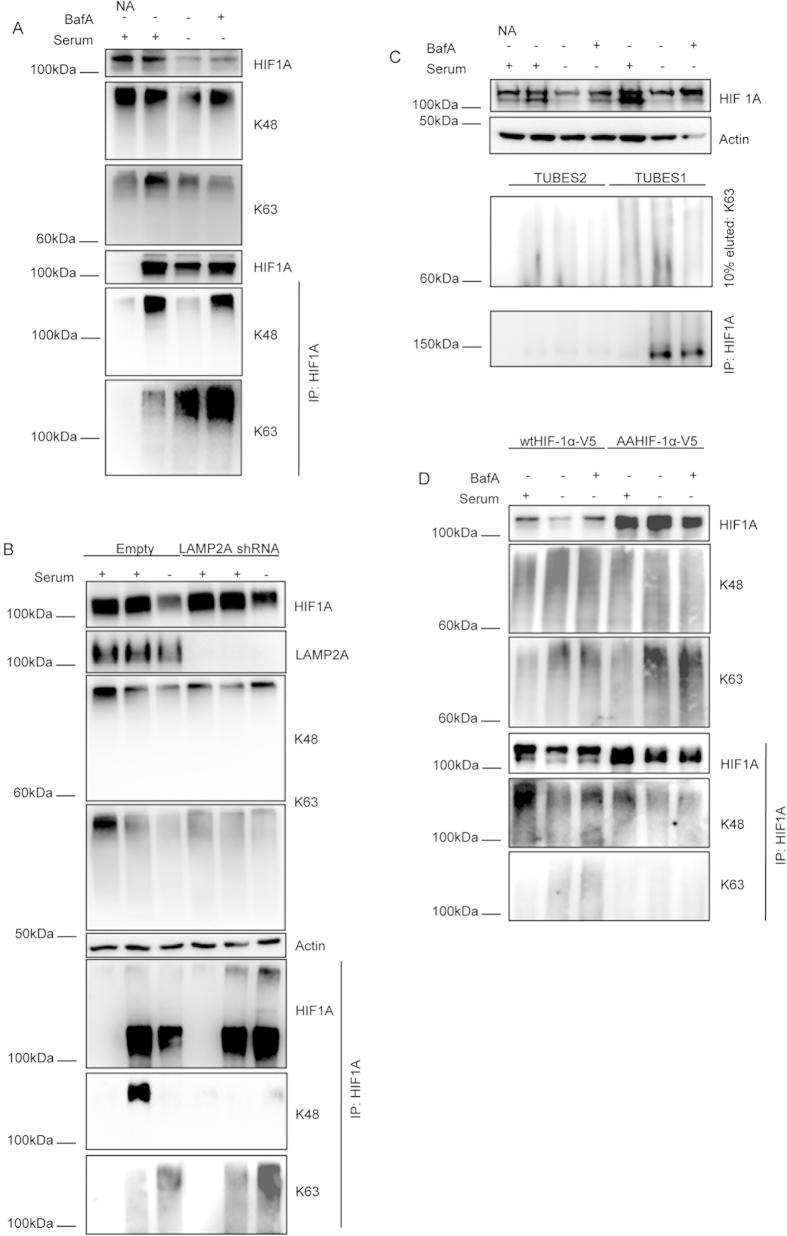
HIF1A is ubiquitinated with K63 linked chains after CMA activation. HeLa and NIH-3T3 cells were incubated in the presence or absence of serum, 300 uM of CoCl_2_, 25 nM of BafA for 6 h. When appropriate cell lysates were immunoprecipitated for HIF1A in denaturing conditions and immunoprecipitates were blotted with antibodies for specific ubiquitin chains. (**A**) Serum removal decreases K48 linked ubiquitination and increases K63 linked ubiquitin conjugation in HeLa cells. (**B**) NIH-3T3 cells were transduced with empty or shRNA containing lentiviral vectors against LAMP2A. Serum removal decreases K48 linked ubiquitination and increases K63 linked ubiquitin conjugation in wild-type cells. LAMP2A depleted cells have increased levels of K63 ubiquitinated HIF1A both in +serum and –serum conditions, when compared to wild-type cells. LAMP2A depleted cells show no detectable levels of K48 ubiquitinated HIF1A. (**C**) HeLa cell lysates were incubated either with TUBEs1 or TUBEs2 enrich samples in ubiquitinated proteins. Those samples were subsequently immunoprecipitated for HIF1A. TUBEs1, but not TUBEs2, samples are enriched with K63 ubiquitin chains and have increased HIF1A protein levels after serum removal. (**D**) HeLa cells were transfected either with wtHIF1A or AAHIF1A tagged with V5. KFERQ mutant HIF1A is unable to increase K63 ubiquitination after serum removal. NA (no antibody). The blots used in the figure are cropped. All the gels have been run under the same experimental conditions.

Having demonstrated that K63 linked chain formation is required for the CMA dependent degradation of HIF1A, we next evaluated the type of ubiquitin chains attached to HIF1A after CMA activation. To address this issue we immunoprecipitated HIF1A in denaturing conditions, to destroy all non-covalent protein-protein interactions, and checked for HIF1A ubiquitination using antibodies for specific ubiquitin chains^29^. The results presented in Fig. 2A show that, after serum removal, ubiquitination of HIF1A by K63 linked chains increased while K48 ubiquitin conjugation to HIF1A decreased (Fig. 2A). Moreover, inhibition of lysosomal proteolysis with BafA resulted in further increase of K63 ubiquitination of HIF1A, while K48 ubiquination reverted to control levels, in the presence of serum (Fig. 2A). This increase in K48 ubiquitination after lysosomal inhibition might just reflect an increase in the levels of immunoprecipitated HIF1A, comparable to control conditions (Fig. 2A). Importantly, in NIH-3T3 cells depleted of LAMP2A, the level of K63-linked ubiquitin chain attached to HIF1A, determined in denaturing conditions, was increased when comparing to the wild-type cells, both in +serum and –serum conditions (Fig. 2B). On the other hand, while both wild-type NIH-3T3 and HeLa cells show a decrease of HIF1A K48 ubiquitination after serum removal, in LAMP2A depleted cells the levels of K48-ubiquitinated HIF1A are virtually undetectable, even in the presence of serum. Altogether this data strongly suggests that CMA inhibition induces the accumulation of K63 ubiquitinated, but not K48 ubiquitinated, HIF1A. To further confirm this data we used an alternative approach, incubating HeLa cell lysates with two different types of GST bound Tandem Ubiquitin Binding Entities (GST-TUBEs), to selectively enrich the samples in ubiquitinated proteins, after which HIF1A was immunoprecipitated in denaturing conditions. TUBEs1 have a 10 fold higher affinity for K63 linked chains than for K48 linked chains, while TUBEs2 have approximately the same affinity for both types of chains. Data in Fig. 2B shows that the levels of HIF1A that co-precipitated with TUBEs1, but not with TUBEs2, increased after serum removal. We have previously shown that HIF1A possesses a KFERQ-like pentapeptide consensus sequence that, once mutated, abrogated HIF1A degradation by CMA^7^. To investigate the involvement of this motif in HIF1A ubiquitination, we immunoprecipitated either wild-type or KFERQ-mutant HIF1A (N529EFKL533 to A529AFKL533) from HeLa cells and evaluated the levels and type of ubiquitin chains attached to HIF1A. The results in Fig. 2C show that only the levels of K63 ubiquitination of wtHIF1A, but not of the mutant HIF1A, increase after serum removal.

To evaluate the subcellular distribution of HIF1A and its co-localization with both the K48 and K63 linked ubiquitin chains we performed confocal microscopy using antibodies against specific ubiquitin chains. Supplementary Fig. 3 and 4 show that serum removal induced a redistribution of K63 chains to the perinuclear region, while no such alteration was observed for K48 chains. Moreover, serum removal led to increased K63 co-localization with HIF1A, present both in the nucleus and the perinuclear region (S.4) whereas co-localization with K48 ubiquitin chains is confined to the nucleus and decreases after serum deprivation.

Thus far, data is consistent with a model where activation of CMA by serum removal acts as a stimulus for the ubiquitination of HIF1A by K63 linked chains, whereas CMA inhibition induces the selective accumulation of K63 ubiquitinated forms of HIF1A. Furthermore, the KFERQ-like motif of HIF1A is essential for the K63 linked ubiquitination of the transcription factor.

### Inhibition of K63 linked ubiquitin chain formation decreases ubiquitin conjugation to HIF1A and interaction of the transcription factor with the CMA receptor LAMP2A

**Figure 3:**
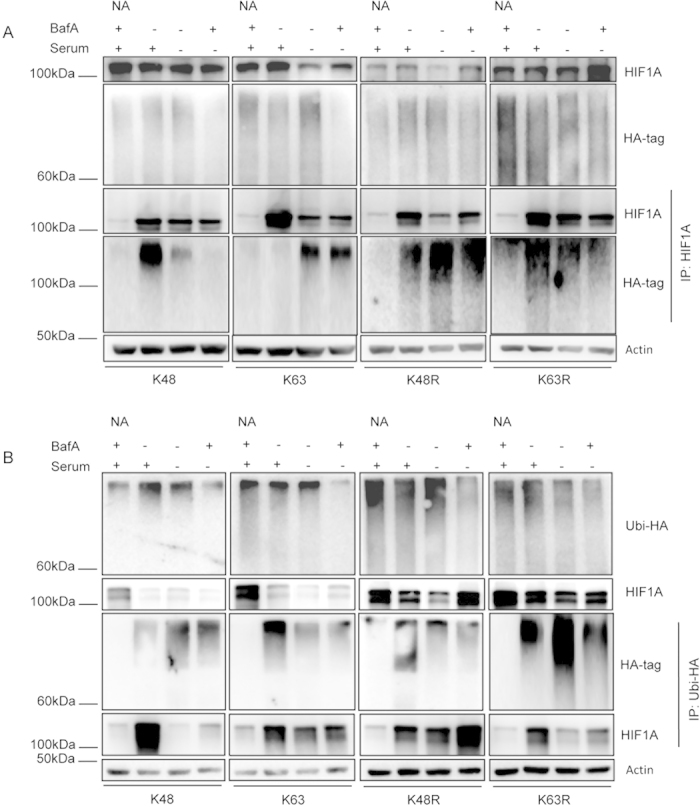
Inhibition of K63 linked ubiquitin conjugation prevents serum deprivation induced ubiquitination of HIF1A. HeLa cells were transfected with plasmids for the mutant ubiquitins tagged with hemagglutinin (HA) and left overexpressing for 48 h. Cells were then incubated in the presence or absence of serum, 300 uM of CoCl_2_ and 25 nM of BafA or for 6 h. Cell lysates were immunoprecipitated with antibodies against HIF1A or HA. (**A**) Immunoprecipitation in denaturing conditions of cell lysates for HIF1A show, after serum removal, an increase in ubiquitination of the transcription factor only in cells overexpressing the K63 and K48R mutants. (**B**) Immunoprecipitation in denaturing conditions of cell lysates for HA tagged ubiquitin show¸ after serum removal, a decrease in co-precipitation of HIF1A with ubiquitin only in cells overexpressing the K48 and K63R mutants. The blots used in the figure are cropped. All the gels have been run under the same experimental conditions. NA (no antibody).

To further confirm the role of K63 ubiquitination in HIF1A degradation by CMA we assessed the amount of HIF1A that was bound to each ubiquitin mutant. Figure 3A shows that, after serum removal, both K48 and K63R mutants, but not the K63 and the K48R mutants, hindered co-precipitation of ubiquitin with HIF1A. In accordance, immunoprecipitation of the ubiquitin mutants showed that, after serum removal, HIF1A decreased its co-precipitation with the K48 and K63R ubiquitin mutants while maintaining it for the remaining ones (Fig. 3B).

**Figure 4:**
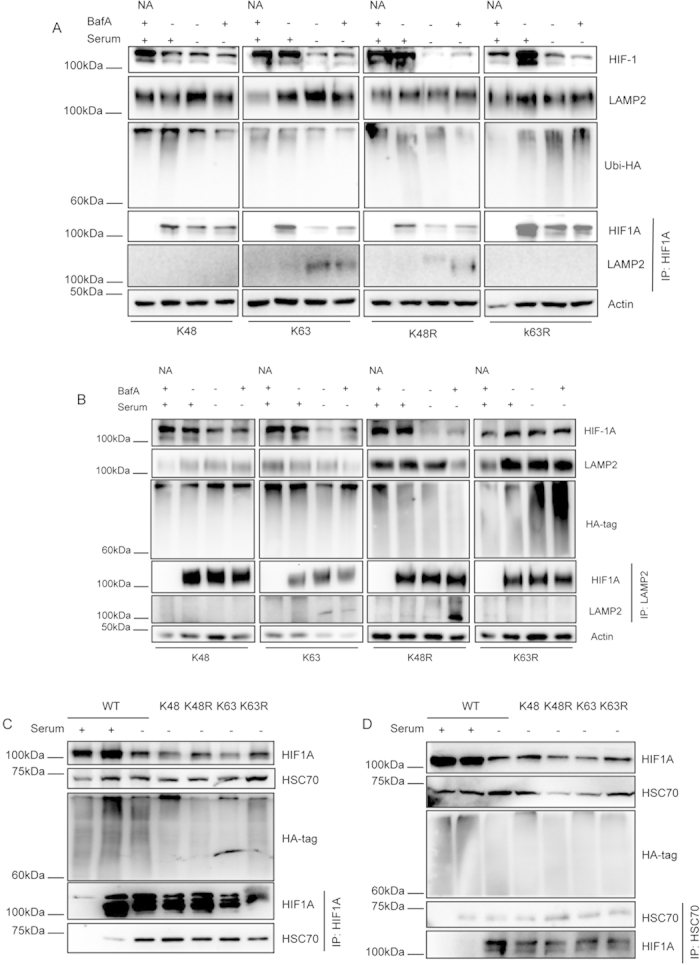
Inhibition of K63 linked ubiquitin conjugation prevents HIF1A interaction with the CMA receptor LAMP2A but not with the chaperone HSC70. HeLa cells were transfected with plasmids for the mutant ubiquitins tagged with hemagglutinin (HA) and left overexpressing for 48 h. Cells were then incubated in the presence or absence of serum, 300 uM of CoCl_2_ or 25 nM of BafA for 6 h. Cell lysates were immunoprecipiatated with antibodies against HIF1A or LAMP2A. (**A,B**) Immunoprecipitation in native conditions of HeLa cell lysates for HIF1A or LAMP2A shows, after serum removal, an increase in the co-precipitation of both proteins with each other only in cells overexpressing the K63 and K48R mutants. (**C,D**) Immunoprecipitation in native conditions of cell lysates for HIF1A or HSC70 shows, after serum removal, an increase in co-precipitation of both proteins with each other. The levels of co-precipitation do not change after the overexpression of the mutant ubiquitins. The blots used in the figure are cropped. All the gels have been run under the same experimental conditions. NA (no antibody).

We had already reported that HIF1A interacts with the CMA receptor LAMP2A and that this interaction was increased after CMA activation^7^. To investigate the role of ubiquitination in modulating HIF1A interaction with LAMP2A we evaluated the effect of overexpressing the ubiquitin mutants in the interaction between HIF1A and LAMP2A. Immunoprecipitation, this time under native conditions, with antibodies against HIF1A and LAMP2A shows that the interaction between LAMP2A and HIF1A is lost only when we overexpress mutants that impair K63 ubiquitin chain formation (Fig. 4A,B).

We had already shown that the KFERQ-motif present in HIF1A is required for the interaction with the chaperone HSC70, a molecular player of the CMA machinery^21^ that is essential for CMA-mediated degradation of the transcription factor^7^. Therefore we proceeded to evaluate whether the type of ubiquitin chains attached to HIF1A determines the interaction with HSC70. To accomplish this objective we evaluated the HIF1A interaction with HSC70 in cells overexpressing either the wild-type or mutated forms of ubiquitin. Immunoprecipitation under native conditions of either HIF1A or HSC70 shows that the proteins co-precipitate with each other, as well as, that serum removal increases their interaction (Fig. 4C,D). Nevertheless, in cells that overexpress the ubiquitin mutants, serum removal does not affect the interaction of HIF1A and HSC70 (Fig. 4C,D).

Overall we demonstrate that the attachment of K63 linked chains to HIF1A is required for its interaction with the CMA receptor LAMP2A but not with the chaperone HSC70.

### Starvation induces HIF1A ubiquitination through K63 linked chains

**Figure 5:**
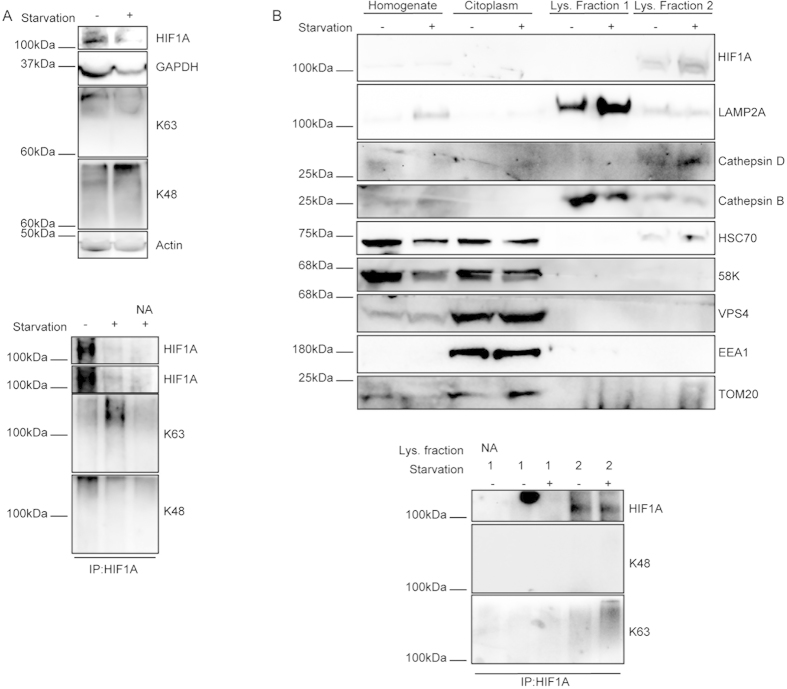
Starvation induces K63 ubiquitin conjugation in HIF1A. (**A,B**) Livers of either fed or 48 h starved rats were homogeneized and, when appropriate, lysosomes were isolated. Homogenates and liver fractions or HIF1A immunoprecipitates were blotted with the appropriate antibodies. (**A**) Liver homogenates from starved animals have lower protein levels of the CMA substrates GAPDH and HIF1A and immunoprecipitation of the transcription factor, in denaturing conditions, show an increase in K63, but not K48, ubiquitin conjugation. (**B**) Isolated liver lysosomes from fraction 2 are enriched in HSC70, LAMP2A, the lysosomal enzymes cathepsin B and D HIF1A. HIF1A enrichment is increased in lysosomes isolated from starved rat livers. Both fractions are devoid of markers for other cellular compartments (58K for Golgi; vps4 for MVBs; EEA1 for early endosomes and TOM20 for mitochondria). HIF1A immunoprecipitated from liver lysosomes show K63 linked, but not K48 linked, ubiquitination of the transcription factor. The K63 ubiquitination of immunoprecipitated HIF1A is increased in liver lysosomes isolated from starved rats. The blots used in the figure are cropped. All the gels have been run under the same experimental conditions. NA (no antibody).

We had previously reported that rats subjected to starvation, known to activate CMA at the organism level^30^, presented higher levels of HIF1A in liver CMA competent lysosomes, although the total levels of HIF1A were reduced in liver homogenates^7^. Therefore, it is reasonable to hypothesize that degradation of HIF1A in the lysosome of starved rat livers requires K63 ubiquitination. To address this question we evaluated the total amount of HIF1A and GAPDH (a known substrate of CMA^31^) as well as the levels and type of ubiquitin chains attached to HIF1A in livers from control and starved rats. Data shows that starvation results in lower levels of both HIF1A and GAPDH (Fig. 5A and S.5A) and that starvation increased K63 ubiquitination and reduced K48 ubiquitination of HIF1A (Fig. 5A). Also, HIF1A accumulates in isolated liver lysosomes enriched in HSC70, that are likely to correspond to CMA competent lysosomes, being this result particularly evident in lysosomes isolated from starved rat livers (Fig. 5B). Strikingly, HIF1A localized in liver lysosomes enriched in HSC70 is ubiquitinated with K63 ubiquitin chains and this ubiquitination increases after starvation, whereas K48 ubiquitination is virtually undetectable (Fig. 5B). These results strongly suggest that K63 ubiquitination and subsequent degradation of HIF1A by CMA is a process that occurs both *in vitro* and *in vivo*.

### STUB1 regulates K63 linked ubiquitination of HIF1A upon CMA activation

**Figure 6:**
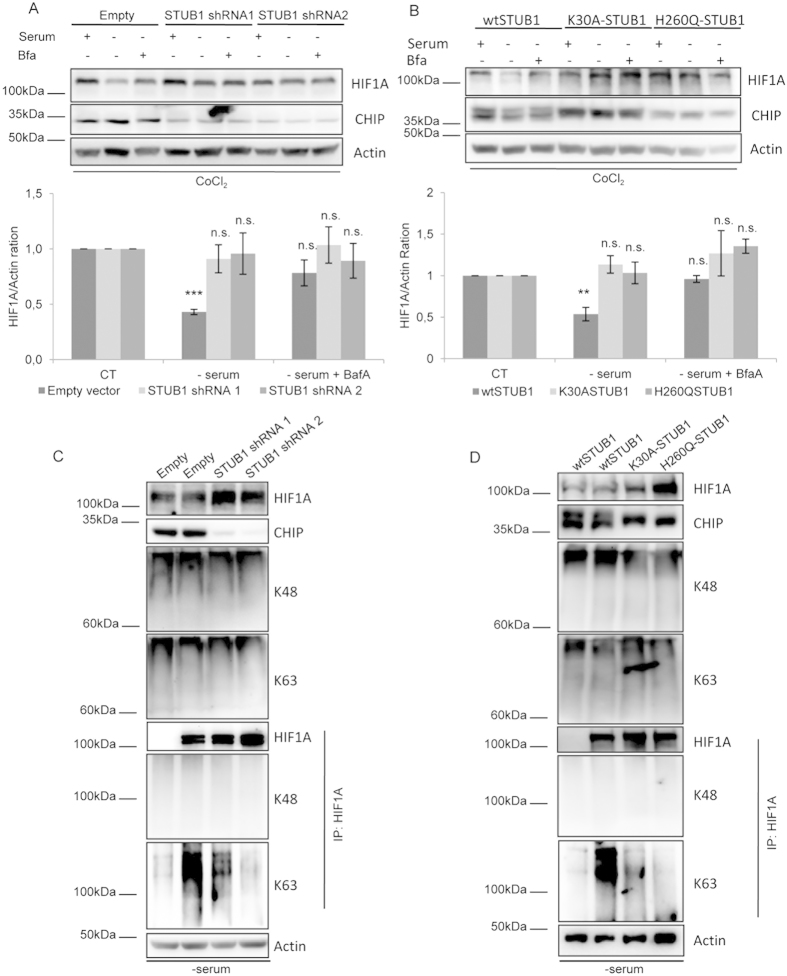
STUB1 is necessary for HIF1A ubiquitination by K63 linked chains and subsequent degradation by CMA. HeLa cells were either transfected with the STUB1 mutants K30A and H260Q and left overexpressing for 48 h or transduced with 2 different shRNA sequences against STUB1 for 72 h. Cells were then incubated in the presence or absence of serum, 300 uM of CoCl_2_ and 25 nM of BafA for 6 h. Also, for the last 4 h, cells were incubated with 50 ug/ml of CHX to inhibit protein synthesis in all samples. (**A,B**) Both STUB1 depleted and K30A or H260Q overexpressing cells are unable to degrade HIF1A after serum deprivation and are insensitive to lysosomal inhibition with BafA. (**C,D**) Both STUB1 depleted and K30A or H260Q overexpressing cells show a decrease in K63, but not K48, ubiquitination after serum removal. The blots used in the figure are cropped. All the gels have been run under the same experimental conditions. NA (no antibody). These results represent the mean ± SD of at least three independent experiments (n.s. nonsignificant; *p < 0.05; **p < 0.01; ***p < 0.001).

Results from our laboratory established that the ubiquitin ligase STUB1 can bind to HIF1A and is necessary for the interaction between HIF1A and LAMP2A^7^. Nevertheless, the question of whether STUB1-mediated ubiquitination of HIF1A is required for CMA degradation remained unexplored. To address this issue we impaired STUB1 activity either by depleting the protein, using shRNA sequences against STUB1, or by overexpressing dominant-negative mutants of the ubiquitin ligase, the STUB1K30A mutant, which is unable to bind chaperones, and the STUB1H260Q mutant, that cannot ubiquitinate substrates. As expected, impairment of STUB1 activity makes HIF1A resistant to degradation and insensitive to the lysosomal inhibitor BafA, after serum deprivation (Fig. 6A,B). Importantly we show that, under serum deprivation, depletion of STUB1 or overexpression of the STUB1 mutants, unable to catalyze protein ubiquitination, results in the loss of K63 ubiquitination of HIF1A (Fig. 6C,D).

Data gathered so far leads us to propose that ubiquitination by K63 linked chains, required for efficient degradation of HIF1A by CMA, is catalyzed by STUB1.

## Discussion

In this study we established, for the first time, that lysine 63 linked ubiquitin chains can target a substrate, in this case HIF1A, for degradation by CMA. Significantly, ubiquitination of HIF1A by K63 chains was also observed in animal tissue after starvation. Our data further shows that both the KFERQ-motif of HIF1A and the ubiquitin ligase STUB1 are essential for K63 ubiquitination and subsequent degradation of HIF1A by CMA.

The results obtained in this study established a further step of specificity regarding the targeting of a substrate for CMA degradation, formerly exclusively attributed to the KFERQ-like motif. The existence of a consensus sequence in a protein that recruits sufficient machinery for its ubiquitination and subsequent degradation creates a parallel to the N-end rule pathway and/or degron sequence recognition^32^,^33^, that determine ubiquitination and subsequent degradation of some substrates by the UPS. In the same context, a specific deubiquitinating enzyme (UCHL-1) that is not a substrate for CMA and that interacts with K63 linked ubiquitin chains was found to be bound to the CMA receptor LAMP2A at the lysosomal membrane^34^,^35^. Although the function of this protein, when interacting with LAMP2A, is still unknown, it is possible that it might act as a receptor for K63 conjugated substrates. In concept, it would be similar to the role of the deubiquitinating enzymes present in the cap of the proteasome, removing the ubiquitin chains prior to substrate degradation^36^.

On the other hand, K63 linked chains are known be able to act as a scaffold for the formation of large protein complexes^37^. In our context, it could be the case that K63 ubiquitination of HIF1A is important in the formation and/or stability of the complex made by HIF1A, HSC70 and assisting chaperones and co-chaperones. In any case, further studies will be required to clarify the role of K63 ubiquitin conjugation in CMA-mediated degradation of HIF1A in particular, but also in CMA in general, since the same concept might be extended to other CMA substrates.

Our lab and others already reported that STUB1 was present in a protein complex with HIF1A, probably through the interaction with HSC70, and was required for CMA degradation of the transcription factor^7^,^22^. In this study we additionally demonstrate that STUB1 catalyzes K63 ubiquitin conjugation to HIF1A that signals subsequent degradation by CMA. Whether this role of STUB1 can be extended to other CMA substrates will also need further research.

Ubiquitination of HIF1A by E3s other than the canonical, oxygen-dependent, VHL-mediated one is not without precedent. Besides STUB1, other examples of HIF1A ubiquitination by alternative E3s are RACK1^38^, HAF^39^, MDM2^40^ and recently TRAF6^41^. Nevertheless, none of these other ubiquitin ligases were reported to induce HIF1A degradation in the lysosome or by CMA. In the case of TRAF6, authors showed that this ubiquitin ligase induced K63 conjugation to HIF1A, with the cardinal difference of stabilizing the HIF1A protein instead of inducing its degradation, implying that HIF1A ubiquitination attributed to both TRAF6 and STUB1 are separated events. Also, we and others had previously shown that STUB1 can mediate proteasomal degradation of HIF1A^22^,^42^. On the other hand, STUB1 is able to ubiquitinate substrates both by K48 and K63 linked ubiquitin chains^43^. This plasticity in ubiquitin chain formation is, at least in part, regulated by the interaction of different E2s with STUB1^43^. Therefore, it is conceivable that different stimuli might induce a specific duo of STUB1-E2 enzymes, able to switch HIF1A between K48 and K63 ubiquitin chain conjugation and, consequently, between the UPS and CMA. The involvement of other ubiquitination machinery in the degradation of HIF1A by CMA will require further study.

In our cellular model, in hypoxic conditions, the inhibition of K63 instead of K48 ubiquitin conjugation stabilized HIF1A protein levels. This implies that K63, but not K48, linked ubiquitination is responsible for HIF1A degradation during hypoxia. The mechanism described in our studies for the degradation of HIF1A by CMA, as well as other alternative mechanism described for HIF1A proteolysis, are likely to impact on a variety of pathophysiological conditions where cells are submitted to prolonged hypoxia (e.g. solid tumor growth, diabetic retinopathy, cardiac infarction). Therefore, it will be of paramount importance to evaluate the relevance of the different HIF1A hypoxia-independent degradation mechanisms in different biological contexts.

## Methods

### Animals and cells

We used male Wistar rats (200 to 250 g). Wistar rats were handled according to the EU guidelines for the use of experimental animals (86/609/EEC). Experiments were approved by the Ethic Committee of the Faculty of Medicine, University of Coimbra, Portugal. We also used the HeLa cells (ATCC, CCL-2) and NIH-3T3 cells kindly provided by Dr. Ana Maria Cuervo.

### Cell culture and treatments

The HeLa and NIH-3T3 cells were maintained in Dulbecco’s modified Eagle’s medium (DMEM; Gibco, 21969-035) supplemented with 10% fetal bovine serum (FBS; Gibco, 1500.064), antibiotics (100 U/ml penicillin and 100 μg/ml streptomycin (Gibco, 15140) and Glutamax (Gibco, 35050). For the experiments cells were incubated in 10% neonatal calf serum (NCS; Hyclone, SH30401.02). When appropriate, cells were treated with the following agents: 300 μM cobalt chloride (CoCl_2_, Sigma-Aldrich, 255599), 10 mM 3-methyladenine (3-MA, Sigma-Aldrich, M9281) 50 nM Bafilomycon A1 (Millipore, 88899-55-2), 6-Aminonicotinamide (6-AN, Sigma-Aldrich, A68203) and Cicloheximide (CHX, Sigma-Aldrich, C-7698). An incubator Nuair N4950E (Nuaire, N4950E) was used to perform the hypoxic treatments (2% O_2_, 5% CO_2_, 37 °C). Transient transfection of cells was performed with lipofectamine 2000 (Invitrogen, 11668-019), according to manufacturer’s recommendations.

### Antibodies and reagents

The following antibodies were used: rabbit anti-HIF1A PA1-16601, dilutions of 1:1000 (western blot); mouse anti HIF1A dilution 1:50 (immunoflourescence) (R&D, MAB1536); goat anti HIF1A dilution 1.1000 (western blot) (Sicgen, AB0112-200); mouse anti-actin, dilutions of 1:2000 (Sigma, AS441); rat anti-Hsc70 clone 1B5, dilution of 1:2000 (Stressgen, ADI-SPA-815); mouse anti-c-myc clone 9E10, dilution of 1:1000 (Zymed-Invitrogen, 18-0176); mouse anti-LAMP2 clone H4B4, dilution of 1:1000 (Santa Cruz Biotechnology, SC756); goat anti-GAPDH dilution 1:2000 (Sicgen, AB0049-200); rabbit anti k48 ubiquitin chains clone APU2 dilution 1:2000 (Millipore, 05-1307); rabbit anti k63 ubiquitin chains clone APU3 dilution 1:300 (Millipore, 05-1308); goat anti STUB1 dilution 1:500 (abcam, ab2482); mouse anti 58K (Sigma, g-2404); rabbit anti LC3 dilution 1:500 (Thermo, PA116931); rabbit anti p62 (CellSignalling, 5114S); rabbit anti vps4 1:500 (Santa Cruz Biotechnology, sc-133122); mouse anti EEA1 1:500 (BD Transduction Laboratories, 14/EEA1); rabbit anti TOM20 1:1000 (Santa Cruz Biotechnology, FL-145); goat anti Cathepsin B (Santa Cruz Biotechnology, S-12); rabbit anti Cathepsin D 1:200 (Genetex, EPR30574) and horseradish peroxidase-conjugated secondary goat anti-mouse (Bio-Rad, 170-6516), goat anti-rabbit (Bio-Rad, 170-6515) and rabbit anti-goat (Bio-Rad, 172-1034), dilution of 1:5000. Alexa Fluor 568-conjugated goat anti-mouse (Invitrogen, A-11019) and green-conjugated Alexa Fluor goat anti-rabbit dilution of 1:100 (Invitrogen, A-11070). Protein G–Sepharose (GE Healthcare, 17-0618-01). Glutathione-Separose (GE healthcare). Polyvinylidene fluoride (PVDF) membranes (GE Healthcare, 10485288). ECL (Bio-Rad, 170-5060).

### Plasmids

For this work we used the following plasmids: pcDNA3.1 c-myc-CHIP wt, pcDNA3.1 c-myc-CHIP K30A, pcDNA3.1 c-myc-CHIP H260Q^44^; pcDNA3 *HIF1A* wt-V5^45^ and pcDNA3 *HIF1A* (*HIF1A*P402A and *HIF1A*P564A)-V5^7^; pRK5-HA-ubiquitin-WT and all ubiquitin mutants, but the K63R mutant, were obtained from Addgene. The K63 ubiquitin mutant was originated by site directed mutagenesis of the pRK5-HA-ubiquitin-WT plasmid.

### Immunoprecitation

Cells were collected from dishes with ice cold PBS using a cell scrapper and centrifuged at 15000 × g for 10 minutes. In all cases pellets were resuspended in 150 μl of lysis buffer (50 mM TRIS-HCl pH 7.4, 150 mM NaCl, 10 mM iodoacetamide, 2 mM PMSF, 20 mM Na3MoO4). For immunoprecipitations in denaturing conditions pellets were resuspended in lysis buffer containing 1% SDS, boiled at 100 °C and sonicated 4 times, 10 s each. After, samples were centrifuged at 15000 × g for 10 minutes, pellets were discarded and sample buffer without detergent was added to a final of 0.2% SDS. For immunoprecipitions in native conditions pellets were resuspended in sample buffer containing 0.5% of NP-40, and sonicated 3 times for 1 s on ice. After, samples were centrifuged and at 15000 × g for 10 minutes and pellets were discarded. All samples were incubated with 2 μg of the antibody against the protein of interest overnight at 4 °C, 30 μl of protein G–Sepharose was added to the sample and incubations proceeded at 4 °C for 2 h. Beads were washed 3 times with lysis buffer containing 0.2% SDS or 0.15% NP-40 respectively, denatured with 2× Laemmli buffer and boiled at 100 °C. Samples were then analyzed by SDS-PAGE. The membranes were blocked with 5% non-fat milk in TBS-T and probed for the proteins of interest.

### Isolation of lysosomal fractions

Male Wistar rats were fasted for 48 h before euthanasia for lysosomal isolation. Lysosomes were isolated using the lysosome isolation kit (Sigma-Aldrich) in a Optiprep density gradient, following step by step the instructions of the technical bulletin. In all cases 6 franctions were isolated after ultracentrifugation of the liver tissue and the acid phosphatase assay (sigma-aldrich) was performed to access the fractions positive for lysosomes. From each sample 2 fractions were isolated that were enriched in acid phosphatase. Samples of the homogenate and and lysosomal fractions, containing 100 ug of protein, were denatured in sample buffer and runned in SDS-PAGE.

### Viral shRNA production and infection

For shRNA targeting of human *STUB1* and mouse *lamp2a*, virus were produced and cells were infected as described before^7^.

### Enrichment of ubiquitinated substrates with GST-TUBEs

For sample enrichment in ubiquitinated substrates cells were scraped off the dishes and collected in ice-cold PBS. Pellets were resuspended in 150 μl of lysis buffer (50 mM TRIS-HCl pH 7.4, 150 mM NaCl, 10 mM iodoacetamide, 2 mM PMSF, 20 mM Na3MoO4, 0.5% NP-40 and protease inhibitor cocktail). Lysates were briefly sonicated and centrifuged at 16,000 × g for 10 min. The samples were incubated overnight at 4 °C with 100 μg/ml of GST-TUBEs (LifeSensors, UM102). Thereafter, 30 μl of protein gluthatione–sepharose 4B (GE Healthcare, 17-0756-01) was added and incubations proceeded at 4 °C for 2 h. Beads were washed 3 times with lysis buffer and pulled-down proteins were eluted with 500 μl of lysis buffer supplemented with 10 mM of reduced gluthatione (Sigma-Aldrich, G4251). Samples were incubated with 2 μg of anti-HIF1A antibody overnight at 4 °C, 30 μl of protein G–Sepharose was added to the sample and incubations proceeded at 4 °C for 2 h. Beads were washed 3 times with lysis buffer, denatured with 2× Laemmli buffer and boiled at 100 °C. Samples were then analyzed by SDS-PAGE. The membranes were blocked with 5% non-fat milk in TBS-T and probed for the proteins of interest.

### Statistical analysis

Data are reported as the means ± standard deviation of at least three independent experiments. Comparisons between multiple groups were performed by one-way analysis of variance test (ANOVA) with Tukey’s multiple comparison tests, using GraphPad Prism 5.0 software (GraphPad Software). For comparison between two groups, the paired t-test was used. In all cases, p < 0.05 was considered significant.

## Author Contributions

J.V.F. and H.G. wrote the manuscript; J.V.F. prepared the figures; J.V.F., H.G. and A.S. designed the experiments; J.V.F., A.S. and J.R.S. performed the experiments and J.V.F., H.G. and P.P. conceived the intial idea of the study. All authors critically revised the manuscript.

## Additional Information

**How to cite this article**: Ferreira, J.V. *et al*. K63 linked ubiquitin chain formation is a signal for HIF1A degradation by Chaperone-Mediated Autophagy. *Sci. Rep*. **5**, 10210; doi: 10.1038/srep10210 (2015).

## Supplementary Material

Supplementary Information
