# Supplementary material for: K63 linked ubiquitin chain formation is a signal for HIF1A degradation by Chaperone-Mediated Autophagy
Source: Sci Rep. 2015 May 11;5:10210. doi: 10.1038/srep10210 (PMC4426689; doi:10.1038/srep10210)
Supplement: Supplementary Information [file srep10210-s1.pdf]

Supplementary Information

K63 linked ubiquitin chain formation is a signal for HIF1A degradation by Chaperone-Mediated Autophagy.

Ferreira JV[1]; Soares A[1]; Ramalho JS[2]; Pereira P[1], Girao H[1]

[1]Center of Ophthalmology and Vision Sciences; Institute for Biomedical Imaging and Life Science (IBILI); Faculty of Medicine; University of Coimbra; Coimbra, Portugal
[2] CEDOC; Faculty of Medicine, New University of Lisbon; Lisbon; Portugal

Supplementary Information Legends

Supplementary Figure 1. HeLa and NIH-3T3 cells degrade HIF1A through CMA and not macroautophagy.(A,B) HeLa and cells were incubated in the presence or absence of serum, 15 mM of 6-AN and 25 nM of BafA for 6 h. Both serum deprivation and 6-AN are known to activate CMA [1] (A) Serum removal decreases HIF1A protein levels, a decrease blocked by lysosome inhibition. (B) 6-AN, a known chemical activator of CMA, decreases HIF1A protein levels. Also, HIF1A flux is increased in the presence of 6-AN. (C,D) HeLa cells were incubated in the presence or absence of serum, 300 uM of $CoCl_2$, 2% O_2 and 25 nM of BafA. (C) HeLa cells incubated in 2% O_2 have decreased HIF1A protein levels after serum removal, a decrease reverted by lysosome inhibition. 3-MA, a specific inhibitor of macroautophagy, inhibited de autophagic flux of LC3 and p62 after serum removal but was unable to do so for HIF1A. (D,E) NIH-3T3 cells were incubated in the presence or absence of 15 mM of 6-AN, serum, 300 uM of 3-MA and transduced with empty or shRNA containing lentiviral vectors against LAMP2A. (D) 6-AN reduces HIF1A, but not LC3-II, protein levels. In LAMP2A depleted cells 6-AN fails to decrease HIF1A protein levels. (E) Serum depletion decreases HIF1A protein levels, a decreased that is not reverted by 3-MA. LAMP2A depleted cells are insentive to serum depletion. (F)Hela cells overexpressing the wild-type and mutant ubiquitins were incubated with 2% O_2. Overexpression of the K48 and K63R mutants, but not of the K48R and K63 mutants, inhibits the degradation of HIF1A after 6 h of serum removal. The blots used in the figure are cropped. All the gels have been run under the same experimental conditions. These results represent the mean ± SD of at least three independent experiments (n.s. nonsignificant; $*p < 0.05$; $**p < 0.01$; $***p < 0.001$).

Supplementary Figure 2. K29 linked chain formation is not important for HIF1A degradation by CMA.

(A) HeLa cells were incubated in the presence of $CoCl_2$ and transfected with plasmids for either wild-type or K29 and K29R mutant ubiquitins tagged with hemagglutinin (HA) and left overexpressing for 48 h. The formation of K29 linked ubiquitin chains is not necessary for HIF1A degradation by CMA. (B) HeLa cells were pre-incubated with $CoCl_2$ or 2% of O_2 for two hours to stabilize HIF1A. After, cells were incubated with CHX for the indicated times while $CoCl_2$ was maintained for the remaining of the experiment. (B) Overexpression of the K48 and K63R mutants, but not of the K48R and K63 mutants, shows a slower decrease in HIF1A protein levels. The blots used in the figure are cropped. All the gels have been run under the same experimental conditions. These results represent the mean ± SD of at least three independent experiments (n.s. nonsignificant; $*p < 0.05$; $**p < 0.01$; $***p < 0.001$).

Supplementary Figure 3. CMA activation induces colocalization of HIF1A with K63 linked ubiquitin chains while decreasing colocalization with K48 linked ubiquitn chains. Confocal microscopy of Hela cells in 2% O_2 for HIF1A (magenta) and either K63 or K48 specific

ubiquitin antibody (green). Serum deprivation increases co-localization of HIF1A with K63 linked chains, but not with K48 linked chains.

Supplementary Figure 4. CMA activation induces colocalization of HIF1A with K63 linked ubiquitin chains while decreasing colocalization with K48 linked ubiquitn chains. HeLa cells were incubated in 2% O_2 and in the presence or absence of serum. Pictures of confocal microscopy of HeLa cells of HIF1A (magenta) and either K63 or K48 ubiquitin chains (green) were taken and analyzed with the ImageJ plug-in for colocalization analysis. Colocalized pixels are shown superimposed to the merge of the magenta and green channels. Serum removal induces an increase in colocalization of HIF1A with K63 linked ubiquitin chains (from 1.05% to 4.21 %) and a decrease in the colocalization of the transcription factor with K48 linked ubiquitin chains (from 6.45% to 1.43%).

Supplementary Figure 5. Quantification of the starvation induced degradation HIF1A and GAPDH and of STUB1 depletion. (A) Livers of either fed or 48 h starved rats were homogeneized. Homogenates were blotted with antibodies against HIF1A and GAPDH. Starvation induces the decrease of the CMA substrates GAPDH and HIF1A protein levels (B) Hela cells were transduced with 2 different shRNA sequences against STUB1 for 72 h. STUB1 was depleted from HeLa cells for ≈70% to ≈90%. The blots used in the figure are cropped. All the gels have been run under the same experimental conditions. These results represent the mean ± SD of at least three independent experiments (n.s. nonsignificant; *$p < 0.05$; **$p < 0.01$; ***$p < 0.001$).

Supplementary References

1 Finn, P. F., Mesires, N. T., Vine, M. & Dice, J. F. Effects of small molecules on chaperone-mediated autophagy. *Autophagy* **1**, 141-145 (2005).

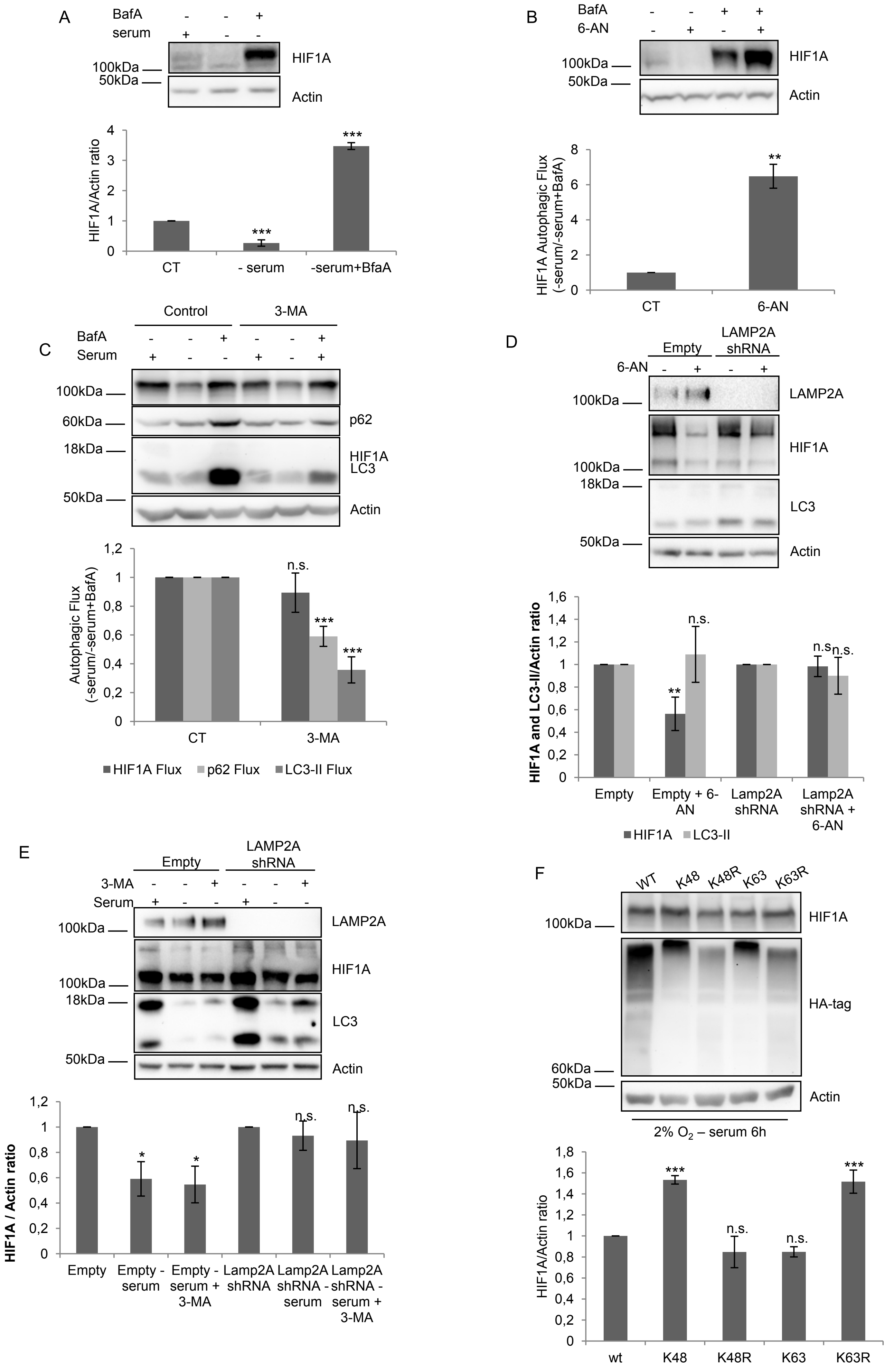

A
BafA - - +
serum + - -
100kDa
50kDa
HIF1A
Actin
HIF1A/Actin ratio
***
***
CT
- serum
-serum+BfaA
B
BafA - - + +
6-AN - + - +
100kDa
50kDa
HIF1A
Actin
HIF1A Autophagic Flux (-serum/-serum+BafA)
**
CT
6-AN
C
Control
3-MA
BafA - - + - - +
Serum + - - + - +
100kDa
60kDa
18kDa
50kDa
p62
HIF1A
LC3
Actin
Autophagic Flux (-serum/-serum+BafA)
n.s.
***
***
CT
3-MA
HIF1A Flux
p62 Flux
LC3-II Flux
D
Empty
LAMP2A shRNA
6-AN - + - +
100kDa
100kDa
18kDa
50kDa
LAMP2A
HIF1A
LC3
Actin
HIF1A and LC3-II/Actin ratio
n.s.
**
n.s
n.s.
Empty
Empty + 6-AN
Lamp2A shRNA
Lamp2A shRNA + 6-AN
HIF1A
LC3-II
E
Empty
LAMP2A shRNA
3-MA - - + - - +
Serum + - - + - -
100kDa
100kDa
18kDa
50kDa
LAMP2A
HIF1A
LC3
Actin
HIF1A / Actin ratio
*
*
n.s.
n.s.
Empty
Empty - serum
Empty - serum + 3-MA
Lamp2A shRNA
Lamp2A shRNA - serum
Lamp2A shRNA - serum + 3-MA
F
WT
K48
K48R
K63
K63R
100kDa
60kDa
50kDa
HIF1A
HA-tag
Actin
2% O2 – serum 6h
HIF1A/Actin ratio
***
n.s.
n.s.
***
wt
K48
K48R
K63
K63R

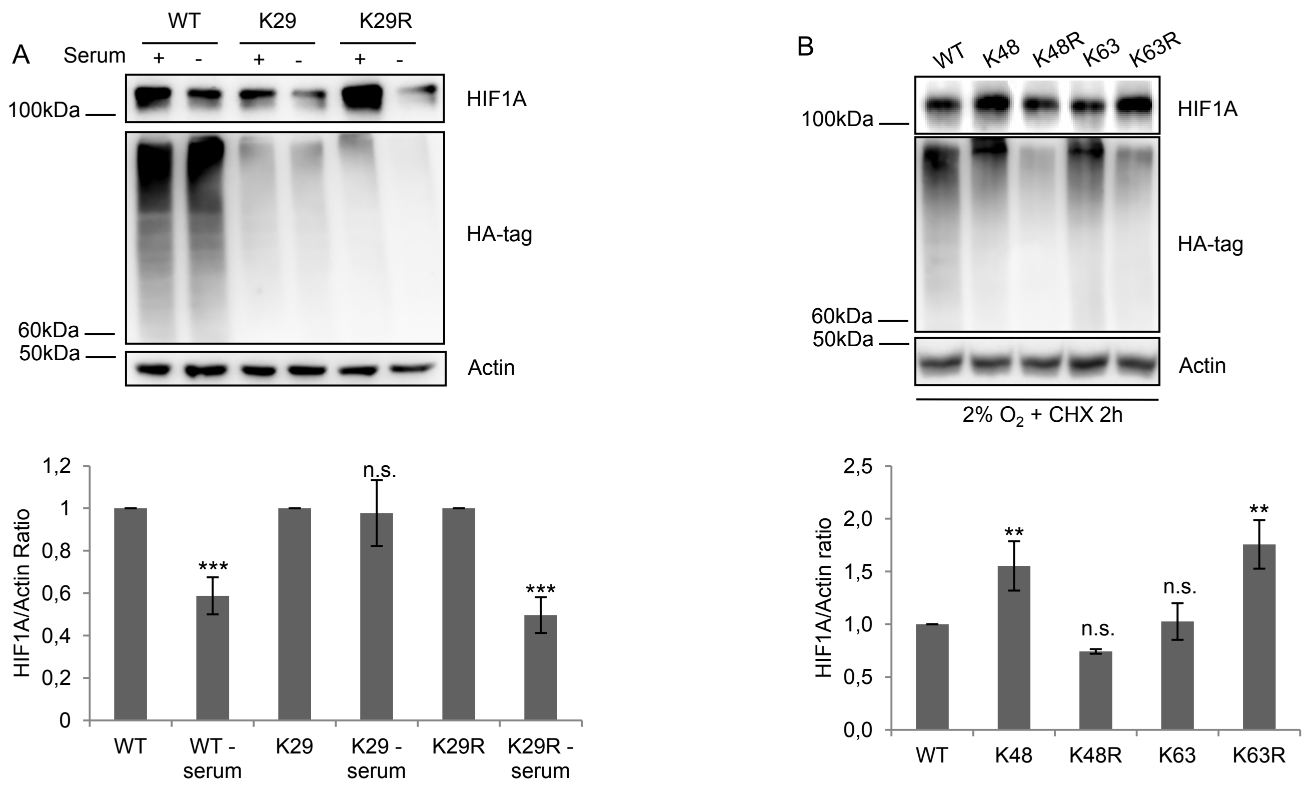

A
WT
K29
K29R
Serum
+
-
100kDa
60kDa
50kDa
HIF1A
HA-tag
Actin
HIF1A/Actin Ratio
1,2
1
0,8
0,6
0,4
0,2
0
n.s.
***
***
WT
WT - serum
K29
K29 - serum
K29R
K29R - serum
B
WT
K48
K48R
K63
K63R
100kDa
60kDa
50kDa
HIF1A
HA-tag
Actin
2% O_2 + CHX 2h
HIF1A/Actin ratio
2,5
2,0
1,5
1,0
0,5
0,0
**
n.s.
n.s.
**
WT
K48
K48R
K63
K63R

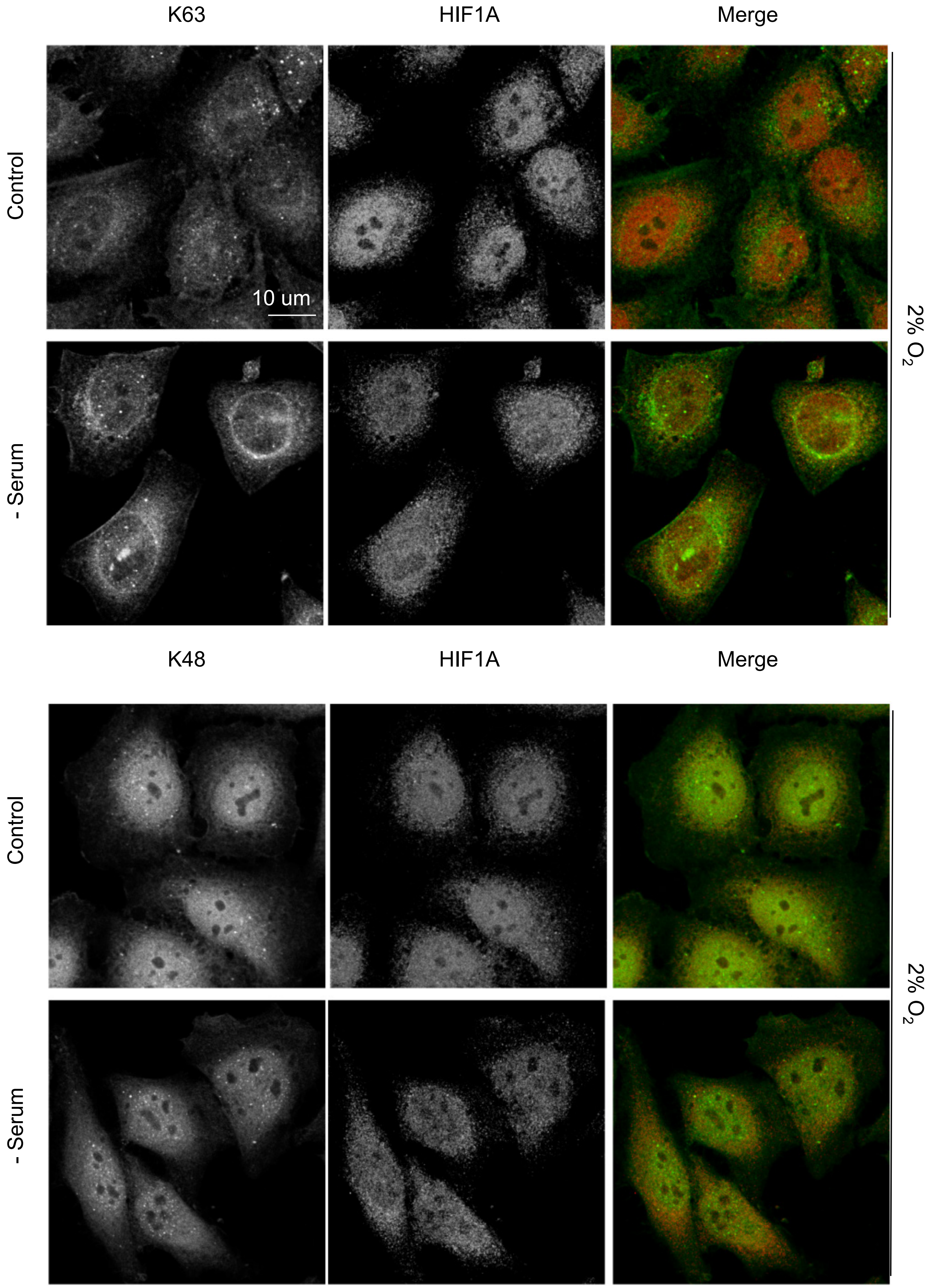
K63
HIF1A
Merge
Control
- Serum
10 um
2% O_2
K48
HIF1A
Merge
Control
- Serum
2% O_2

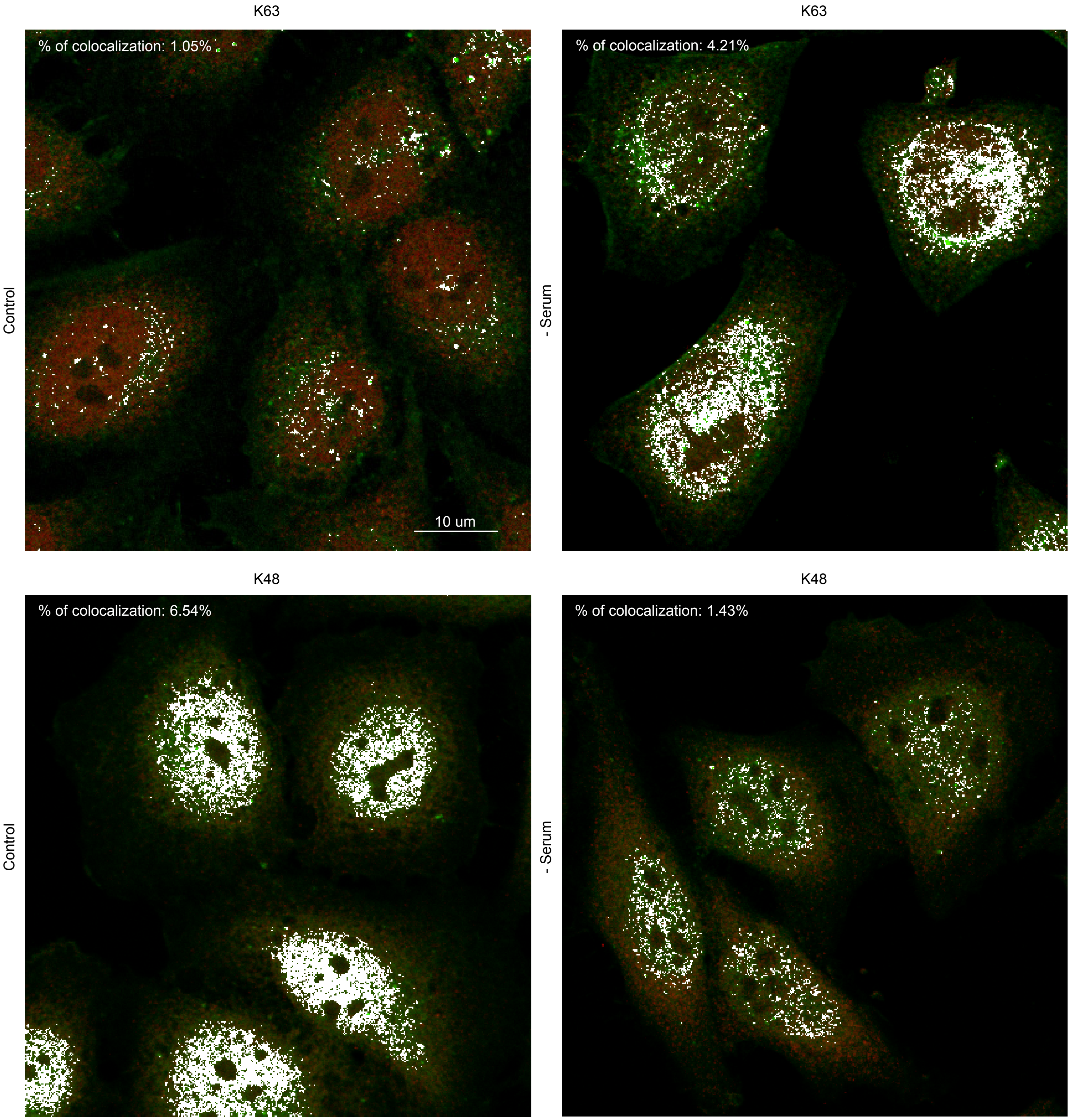
K63
K63
% of colocalization: 1.05%
% of colocalization: 4.21%
Control
- Serum
10 um
K48
K48
% of colocalization: 6.54%
% of colocalization: 1.43%
Control
- Serum

A

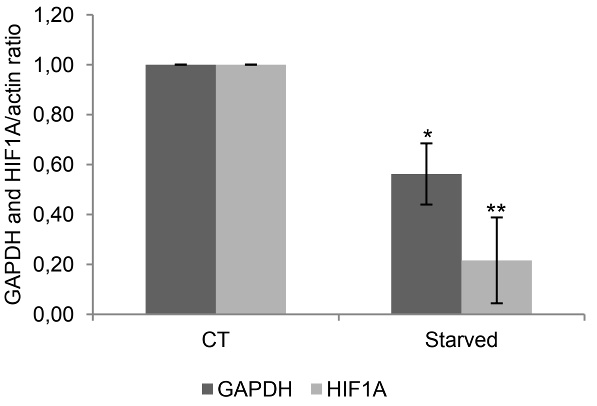

GAPDH and HIF1A/actin ratio
1,20
1,00
0,80
0,60
0,40
0,20
0,00
*
**
CT
Starved
GAPDH
HIF1A

B

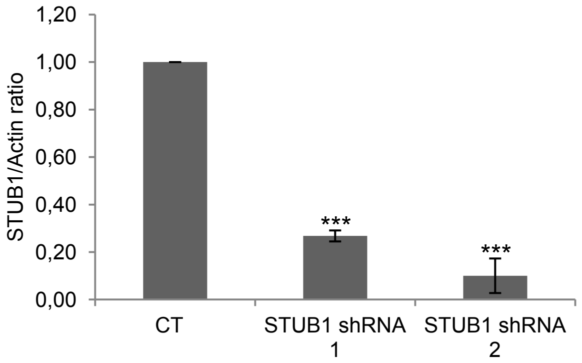

STUB1/Actin ratio
1,20
1,00
0,80
0,60
0,40
0,20
0,00
***
***
CT
STUB1 shRNA 1
STUB1 shRNA 2
